# Doctors' Cars at the Motor Show.—II

**Published:** 1909-01-02

**Authors:** 


### Motoring Notes.

### DOCTORS' CARS AT THE MOTOR SHOW.—II.

There still remain several cars which, owing to lack of space, have not yet been alluded to, but are well worthy of the attention of the medical man.

Phœnix Motors, Ltd., one of the oldest firms of motor manufacturers, whose famous tricar of some years ago will no doubt be remembered, showed two cars likely to suit a doctor. One is a two-cylinder 8 h.-p. car, the other a two-cylinder 10-12 h.-p. Both have a plate clutch, metal to metal brakes, epicyclic gear, and transmission by chain. These chains are, however, protected by long covering pieces which extend beneath each and effectually prevent soiling by mud and dirt. Accumulator ignition is adopted on both models, and sight gauges are fitted to the oil and petrol tanks—a feature that I should be pleased to see other manufacturers adopt. The price of the 8 h.-p. with two-seated body is £140, and that of the 10-12 h.-p. with four-seated side entrance body £235.

The Riley Cycle Co., of Coventry, are this year putting on the market two small cars of 9 h.-p. and 10 h.-p. The 9 h.-p. car is one that has given satisfaction to many users for the past two or three years, and does not reveal many changes. The 10 h.-p. car, however, is a new introduction. It has engine-driven pump lubrication, thermo-syphon water circulation, the Riley patent gear-box of the ever-in-mesh variety, and accumulator ignition. Both cars have twin-cylinder engines of the "V" type. Messrs. Riley showed their detachable and interchangeable wheel, wire or wood pattern, which can be placed or taken off any axle in a couple of minutes. The price of the 9 h.-p. car is £168, with two-seated body, and that of the 10 h.-p. is £200.

Messrs. Osborne and Lord, of Great Marlborough Street, W., showed the well-known 8 h.-p. Gregoire. This car has two cylinders with the valves all on one side, thermo-syphon cooling with very large pipes, and leather-lined clutch with a pressed steel cone. High-tension magneto ignition is employed, and with a two jet carburettor and forced lubrication this car is quite up to date in every respect. I might add that the differential casing is divided horizontally so that the top half can be easily removed for inspection of the bevel gear.

Messrs. Smeddle and Kennedy, of Newcastle-on-Tyne, had one of the novelties of the show in the small-car class. The car is called the S.K. Simplex and it is utterly impossible in the space at my disposal to attempt to describe all the very clever mechanical devices in this car. I believe the main idea of the designers is to arrange and plan things so that the average user without much mechanical knowledge should be actually able to take the whole chassis to pieces and then to refit it. The engine is of 10 h.-p. and of the two-cylinder type, all the valves are overhead, the automatic carburettor is bolted directly on to the inlet ports without any piping intervening, and the flywheel has fan arms and forms the outer member for an internal expanding metal clutch. The complete car with two-seated body sells for £210. The car was considered most original, the only things that followed standard practice being the frame and the radiator; yet I believe there was nothing that an engineering expert could find fault with.

Messrs. Singer, of Coventry, are selling a two-cylinder 7-9 h.-p. car which exhibits all the Singer nicety of finish and exactness of detail. With accumulator ignition, leather cone clutch, gate change-speed gear, and two-seated body it costs £210.

Messrs. Morgan and Co., of Long Acre, W.C., showed a 7 h.-p. two-cylinder Adler car. Some readers may remember that the Adler car gained considerable renown by winning two classes in the 2,000 miles Touring Car Trials. This car, like the Humber, possesses two separate ignitions. The carburettor is automatic, circulation by pump driven by pinions, and with its Morgan two-seated body must be considered cheap at £180.

Chenard-Walcker Motors, Ltd., exhibited a very exceptionally finished 8-9 h.-p. car which was unique among all the small cars at the show in so much as it possessed four forward speeds. The drive on the fourth speed is direct. The carburation is automatic, as is also the lubrication, and thermo-syphon cooling is adopted. The ignition is by high-tension magneto, and the clutch is of the leather to metal type With a smart two-seated body the price is £190.

I think I have now described all the cars that are likely to suit the medical man, and in conclusion will give my impression of the general trend of design of which the late show gave evidence. In regard to the frame the pressed steel variety predominates, although armoured wood and tubular types are favoured by many firms. There seems to be no doubt that the gear-driven car will in the future practically hold the field. Many manufacturers who formerly used the chain drive have now abandoned it, and there are no recruits to this system. The piston stroke in small cars is generally lengthened, and in addition to this there is a tendency to reduction of the cylinder diameter. The area of valve heads has been increased and lubrication is almost universally of the forced feed type, the oil being driven by means of a pump directly on to the bearings. Thermo-syphon cooling is largely in evidence, and many large firms who hitherto were strong adherents of the pump have now become converts to this system. In regard to ignition the high-tension magneto is to be seen everywhere, and seems to be rapidly ousting both the accumulator and coil and the low-tension variety. The sliding gear seems universal with the exception of a few cars in which the epicyclic gearing is used. Finally, I may say that prices are somewhat lower, but it cannot be said that quality has been reduced also, as there is no doubt that the workmanship and mechanical design of the cars have very generally been considerably enhanced.

"Viator."

